# Supplementary material for: Prevalence and pattern of adverse events following COVID-19 vaccination among adult population in Sokoto metropolis, northwest, Nigeria
Source: PLoS One. 2025 Mar 12;20(3):e0277585. doi: 10.1371/journal.pone.0277585 (PMC11903040; doi:10.1371/journal.pone.0277585)
Supplement: S1 Questionnaire — (DOCX) [file pone.0277585.s002.docx]

**QUESTIONNAIRE ON PREVALENCE AND PATTERN OF ADVERSE EVENTS FOLLOWING COVID-19 VACCINATION AMONG ADULT POPULATION IN SELECTED HEALTH FACILITIES IN SOKOTO METROPOLIS, NORTH-WEST, NIGERIA.**

.

Dear Sir/Ma,

We are conducting a research is to assess the prevalence and pattern of adverse events following covid-19 vaccination among adult population in selected health facilities in Sokoto metropolis. Kindly respond to the questions in the questionnaire to the best of your knowledge and ability. All information obtained shall be used for the purpose stated herein and shall be treated with utmost confidentiality; it is therefore, expected that you will give true answers of what you know.

We appreciate and thank you for your time.

Yours faithfully,

- Habibullah Adamu
- Adamu Ahmed Adamu
- Sufiyanu Lawal
- Akilu Muhammad Sani
- Ishaka Alhaji Bawa

**CONSENT**

Do you agree to participate in this study? (a) Yes  (b) No 

Date of data collection: ______________________

1. Questionnaire No:________________________

**SECTION A: SOCIODEMOGRAPHIC CHARACTERISTICS**

1. Age (years):……….
2. Sex:

(a) Male 

(b) Female 

1. Religion:

(a) Christianity 

(b) Islam 

(c) Others  (specify) : …………………………..

1. Tribe:

(a) Hausa/Fulani

(b) Igbo

(c) Yoruba

(d) Others  (specify): …………………….

1. Marital status:

(a) Married

(b)Divorced 

(c) Widow

(d) Single

1. Occupation:

(a)Health worker

(b) Businessman/businesswoman

(c) Self employed

(d) Farmer

(e) Student

(f) Others (specify)……….

**SECTION B: PREVALENCE AND PATTERN OF AEFI FOLLOWING COVID-19 VACCINATION**

8. Did you experienced any reaction following covid-19 vaccination?

(a) YES 

(b) NO 

**9.** If YES to question 8, which of the following reactions did you experience? (please you can select more than one option)

(a) Fever 

(b) Headache

(c) Body weakness 

(d) Pain at the site of injection

(e) Rash 

(f) Muscles pain 

(g) Cough/catarrh

(h) Others (specify)……….

10. When did you experienced the reaction following vaccination?

1. Immediately
2. Within 2-3 days
3. After 1week
4. After 1 month
5. Others(specify)………..

11. For how long have you experience the reaction?

1. Few minutes
2. Few hours
3. 1 days
4. 2 days
5. Others(specify)…………

12. How severe was the reaction?

1. Mild
2. Moderate
3. Severe
4. Very severe

13.If severe, were you admitted in health facility as result of the AEFI?

(a). Yes  (b). No

14. What did you do following the AEFI?

1. Sought medical attention in a health facility
2. Took drugs
3. Nothing

15. If you have experienced AEFI following COVID 19 vaccination, did you notify the vaccination team of the AEFI?

(a). Yes (b). No

**SECTION C: FACTORS ASSOCIATED WITH ADVERSE REACTIONS FOLLOWING COVID-19 VACCINATION.**

**14.** Do you have any underline medical condition?

1. YES 
2. NO 

**15**. If YES to question 14, which of the following medical conditions do you have? (Please you can tick more than one option)

1. Hypertension 
2. Diabetes mellitus 
3. Asthma 
4. Heart disease 
5. Kidney disease
6. Pregnancy 
7. Others (specify)………… (if there is any)

16. If NO to question 14, which of the following do you attribute to the reaction experienced?

1. Vaccine over dose 
2. Expired vaccine 
3. Given by inexperienced health worker 
4. Allergy to the vaccine 
5. I don’t know 

17.Did you experienced any reaction to vaccine in the past?

(a) YES 

(b) NO 

18. Did you experience any drug reaction in the past?

(a) YES 

(b) NO 

19. Are you allergic to anything other than drug or vaccine?

(a) YES 

(b) NO 

20. Do you have any family history of reaction following vaccination?

(a) YES 

(b) NO 

21. Do you have any family history of drug allergy?

(a) YES 

(b) NO 

22. Do you smoke cigarette?

(a) YES 

(b) NO 

23. Do you consume alcohol?

(a) YES 

(b) NO 

THANK YOU FOR TIME AND RESPONSE
