# Supplementary material for: Prevalence and pattern of adverse events following COVID-19 vaccination among adult population in Sokoto metropolis, northwest, Nigeria
Source: PLoS One. 2025 Mar 12;20(3):e0277585. doi: 10.1371/journal.pone.0277585 (PMC11903040; doi:10.1371/journal.pone.0277585)
Supplement: Attachment — Submitted filename: Response to Reviewer - 6.docx [file pone.0277585.s012.docx]

**`REVIEWERS’ COMMENTS AND ACTIONS TAKEN BY AUTHORS - 6**

| SN | **COMMENTS/OBSERVATIONS OF REVIEWER** | **ACTION TAKEN** |
| --- | --- | --- |
|  | **Title** |  |
|  |  |  |
| A | **Abstract** |  |
|  |  |  |
| B | **Introduction** | |
|  | 1. Nil | Nil |
| C | **Materials and method** | |
|  | Nil | 1. Nil |
| D | **Results** | |
|  |  |  |
| E | **Discussion** | |
|  | It would also be important, as already indicated in the conclusions, to indicate how the information to be given to the patient is useful for compliance and fairness | Done |
| F | **Conclusion/recommendation** | |
|  |  |  |
| G | **Reference list** | |
|  | Nil |  |
|  | **Supporting document** | |
|  |  |  |
