# Supplementary material for: Prevalence and pattern of adverse events following COVID-19 vaccination among adult population in Sokoto metropolis, northwest, Nigeria
Source: PLoS One. 2025 Mar 12;20(3):e0277585. doi: 10.1371/journal.pone.0277585 (PMC11903040; doi:10.1371/journal.pone.0277585)
Supplement: Attachment — Submitted filename: Response to Reviewer.docx [file pone.0277585.s007.docx]

**RESPONSE TO REVIEWER**

| SN | **COMMENTS/OBSERVATIONS OF REVIEWER** | **ACTION TAKEN** |
| --- | --- | --- |
|  | 1. Need for statistical significance to support result findings 2. Contradictory statements in the result and conclusion | 1. Respective p-values inserted 2. Contradiction rectified; it is supposed to be presence of underlying disease not absence of… |
|  | **Introduction** | |
|  | Nil | NA |
|  | **Materials and method** | |
|  | 1. The duration/ time period of conducting the survey, inclusion or exclusion criteria should be include 2. To define HFs 3. Include approval number of each ethics committee 4. Please provide additional details regarding how this consent was documented and witnessed, and state whether this was approved by the IRB | 1. Duration/time period indicated. The inclusion/exclusion have been stated already; stated in the 3^rd^ paragraph of material and method section 2. HFs defined 3. Approval number for each committee included 4. Detail provided in the revised manuscript |
|  | **Result** | |
|  | 1. The author mentioned that 230 participants were included in the study. In Table 1, the sum of the Age sub-group shows 231 2. Figure 1: The total number of respondents is shown as 231 3. Figure 2: Y axis needs label 4. Table 2: Number of AEFIs experienced respondents is shown 184 (n). There no explanation why this number decreased from 230 to 184 5. Table 3: Under In the middle column, the total respondents equals 231 (instead of 230). In the right most column (Test Statistic) the box is left empty 6. The authors have designated the AEFI as mild, moderate and severe. But they did not define the basis of this classification | 1. The frequency has been corrected accordingly; it was a typographical error while inputting values in the table 2. Total number corrected 3. Y-axis labelled 4. The explanation is that, the total number of respondents is 230, out of which 184 experienced AEFI whereas 46 did not. So table 2 only focused on those who experienced AEFI because its answering the question of pattern of AEFI experienced 5. All affected frequencies, percent, test-statistic have been corrected after re-running the bivariate analysis 6. It was classified based on respondents’ perception of the AEFI |
|  | **Conclusion/recommendation** | |
|  | To restrict recommendations to study findings | Done |
|  | **Reference list** | |
|  | 1. To complete some references 2. Doi for reference No. 4 is incomplete 3. To provide web address for reference no. 11 4. To correct reference list no.12 | 1. References completed 2. Doi completed 3. The data was sought directly from the Department of Planning Research and Statistics of the NPHCDA (offline) 4. Corrected; however, K. Park is the only author, no editor |
