# Supplementary material for: Prevalence and pattern of adverse events following COVID-19 vaccination among adult population in Sokoto metropolis, northwest, Nigeria
Source: PLoS One. 2025 Mar 12;20(3):e0277585. doi: 10.1371/journal.pone.0277585 (PMC11903040; doi:10.1371/journal.pone.0277585)
Supplement: Attachment — Submitted filename: PONE-D-22-29819 Review report v-2.docx [file pone.0277585.s006.docx]

**Revision-2 PONE-D-22-29819**

**PLOS ONE 2023.05.14**

**Initial recommendation to the Editor:** The manuscript can be accepted with minor revision addressing the following comments to the authors.

**To the authors:**

Thank you for properly addressing the previous queries. Here, during careful checking the manuscript I have found some more areas to be clarified by the authors. I guess you would find these as minor revision.

#1 Figure 1. Y-axis is missing label. Even the values are inserted, it is better to add y-axis label.

#2, Page 11, “N= Total population of adults who received COVID-19 from the 3 selected health facilities (HFs) = 1300”

I think instead of ‘COVID-19’ it will be ‘COVID-19 vaccine’

#3, a) Table 3: In the last column, the symbol is missing, probably. It would be ‘chi-square’. Pease ensure the symbol is typed correctly without any missing.

b) It is not clearly mentioned in the Table by which statistical parameters were determined to estimate the association. Please confirm whether you used ‘OR’ or ‘Chi square’. Then confirm the findings of statistical analysis of the data shown in this Table 3.

**Reviewer: Dr. Md. Mustafizur Rahman**
