# Supplementary material for: Prevalence and pattern of adverse events following COVID-19 vaccination among adult population in Sokoto metropolis, northwest, Nigeria
Source: PLoS One. 2025 Mar 12;20(3):e0277585. doi: 10.1371/journal.pone.0277585 (PMC11903040; doi:10.1371/journal.pone.0277585)
Supplement: Attachment — Submitted filename: Response to Reviewer - 2.docx [file pone.0277585.s009.docx]

**REVIEWERS’ COMMENTS AND ACTIONS TAKEN BY AUTHORS - 2**

| SN | **COMMENTS/OBSERVATIONS OF REVIEWER** | **ACTION TAKEN** |
| --- | --- | --- |
| A | **Abstract** |  |
|  | 1. Need for statistical significance to support result findings 2. Contradictory statements in the result and conclusion | 1. Respective p-values inserted 2. Contradiction rectified; it is supposed to be presence of underlying disease not absence of… |
| B | **Introduction** | |
|  | 1. Background, problem statement, rationale and hypothesis should be provided | 1. Done |
| C | **Materials and method** | |
|  | 1. To organize the methodology and include headings such as eligibility criteria, inclusion and exclusion criteria. 2. Please explain the study design in detail. 4-As the study is cross-sectional, kindly provide a Strobe statement and strobe checklist. 3. Why was the GPower 3.1 tool used to determine the sample size? What were the advantages compared to Power and Sample Size Calculation (PASS) or OpenEpi? Justify the method of calculation. 4. The total population of those who received COVID-19 vaccine at the time of the study was less than 10,000- please justify this figure and the relatively small sample size requirement estimate. 5. Rephrase the estimate of 90% and the statement "Using the above two formulae, sample size of 207 participants was estimated. A response rate of 90% was anticipated, thus the final sample size (nf) was adjusted to 230, by dividing the calculated sample size (207) by the anticipated response rate (0.9); therefore, 230 study participants were enrolled into the study".   Provide references. Why did you estimate it and why did you adjust it? Please justify rational and provide some reference on your methodology. Please provide with minimum 3-4 reference on rational about estimation of 90%? Did you estimate before the study starts and why 90%? its a high number. Please justify in details with reference   6. Please include questionnaire administration as a subheading and support the pre-test statements with references. Please provide provide the questionnaire as supplementary file. 7. Please explain and rewrite clearly the following statement, "The questionnaire was pretested among adult men and women who received COVID-19 vaccines from PHC Yar Akija. Necessary amendments were made thereafter, and the instrument was found valid. Personnel used for data collection comprised the researchers with the help of three research assistants. Data collection was done using the questionnaire described above. The questionnaire was designed and deployed to a web-based account created on [https://kobo.humanitarianresponse.info](https://kobo.humanitarianresponse.info/). The deployed questionnaire was then accessed by downloading Open Data Kit (ODK) app on Android devices and was used for data collection. " 8. Please elaborate on the tool used, its benefits (web based?), and provide information on PHC Yar Akija's population demographics. 9. Please add the heading, "statistical analyses". The current provided limitation does not address all bias. Please provide in detail sampling bias, selection bias, recall bias, measurement bias. Please provide timeline of data collection. | 1. Subheadings included 2. Study designed explained; 3. Sample size was calculated manually using the Cochran formula; detailed calculation has been given and the formulae used were well referenced, including the modified Cochran formula which is used in calculating sample size when the size of the target population is not so large (<10,000); this is an acceptable method of calculating size. 4. In Nigeria, there is a COVID-19 register at each COVID-19 vaccination site where records of all those who received the vaccine are kept; the total number of the recipients in the register was not up to 10,000 (i.e. in the three health facilities selected for the study. This is not surprising considering the fact that vaccine hesitancy has been an issue in Nigeria even before COVID-19 outbreak; this perhaps explains the relatively small number of those vaccinated. healDuration/time period indicated. The inclusion/exclusion have been stated already; stated in the 3^rd^ paragraph of material and method section 5. Statement rephrased and reference provided   Detailed explanation given (with references)   6. Done 7. Explanation given 8. Benefits of using ODK App has been given and additional information on PHC Yar Akija has also been provided 9. Detail on handling bias is provided (see study strength) |
| D | **Result** | |
|  | 1. Figure 1. Y-axis is missing label. Even the values are inserted, it is better to add y-axis label. 2. Page 11, “N= Total population of adults who received COVID-19 from the 3 selected health facilities (HFs) = 1300” I think instead of ‘COVID-19’ it will be ‘COVID-19 vaccine’ 3. a) Table 3: In the last column, the symbol is missing, probably. It would be ‘chi-square’. Pease ensure the symbol is typed correctly without any missing. b) It is not clearly mentioned in the Table by which statistical parameters were determined to estimate the association. Please confirm whether you used ‘OR’ or ‘Chi square’. Then confirm the findings of statistical analysis of the data shown in this Table 3 | 4. Y-axis label added 5. Corrected; vaccine added 6. Test statistic (Pearson chi square) defined below the table; Pearson chi square test was used to test for association (with the respective p-values) |
| E | **Discussion** | |
|  | Rewrite sections of the discussion and compare with findings of available literature | All reported findings have been compared with other findings; implications of findings also discussed.  Additional references have also been given |
| F | **Conclusion/recommendation** | |
|  | Nil | Nil |
| G | **Reference list** | |
|  | Nil |  |
