# Supplementary material for: Prevalence and pattern of adverse events following COVID-19 vaccination among adult population in Sokoto metropolis, northwest, Nigeria
Source: PLoS One. 2025 Mar 12;20(3):e0277585. doi: 10.1371/journal.pone.0277585 (PMC11903040; doi:10.1371/journal.pone.0277585)
Supplement: Attachment — Submitted filename: adverse events following COVID-19 vaccination.docx [file pone.0277585.s008.docx]

**Abstract**

Title: "an" adult population

Background: "As all vaccines are associated with some adverse reactions (ARs)" - to be removed from objectives.

Methods: COVID-19 vaccine"s"

Results: "M"ajority

Conclusion: Remove comma before ","with body weakness

**Background:**

1. Line 1 Severe acute respiratory syndrome ~~the~~ coronavirus

2. Line 2 ~~ongoing~~ pandemic

3. Please update the contents of para 2 and 3 so as to reflect current scenario OR those paragraphs can be removed entirely.

*Till date, there is no known cure for COVID-19, thus control*

*measures are largely preventive in nature, and these include pharmacological and nonpharmaceutical interventions (NPIs) such as regular use of face masks, use of hand sanitizers,social distancing, travel restrictions, partial or complete lockdowns, and use of vaccines...............*

4. Remove the phrase "Problem statement". The para which states why a study in that chosen state helps to fill in a current knowledge gap, should tie in with the preceding para. It is there, please strengthen the relation between problem statement and rationale.

5. Remove the phrase "Rationale". The contents need not be in a new paragraph.

6. Remove "Study hypothesis" and rephrase contents as, "We hypothesized that......"

**Methods:**

**1**. Move around the contents so they fall under the specific subheadings. Use subheadings "Study design and sampling", "Study instruments", Inclusion and exclusion criteria", "Enrollment"

2. Please provide as supplementary material a completed checklist stating page numbers where each of the criterion was fulfilled https://www.strobe-statement.org/checklists/

3. Rephrase so it reflects the past actions of the research team involved.

"So if a researcher anticipates that up to 90% of the subjects will complete

the questionnaires, he should divide the sample size by 90% (i.e. 0.9)."

4. The following content to be reorganised and made clearer. Currently unclear who made the calls, as there was a mention of research assistants in the preceding para.

"Data was collected by administering the questionnaire via personal phone calls to all the selected participants whose phone numbers could be retrieved from the COVID-19 register. Before administering each questionnaire, the research assistants explained the purpose of the research to the respondent and sought their consent to participate. The overall process of recruitment of study participants and collection of data lasted for about 7-8 weeks (between March and May 2022).
5. The Level of statistical

6. Rephrase, "confidentiality of the information provided by the participants was

also assured."

7. Study limitations to be moved to end of discussion section.

**Results:**
1. Please recalculate response rate using initial number eligible for inclusion, then excluded due to missing phone numbers, approached for interview but did not give consent etc.

2. practice"d" Islam as "their" religion

3. Figure 1 can be removed as it is described in text.

4. Rephrase "On bivariate analysis using Pearson chi square test..

5. Logistic regression findings: both presence of comorbidities and hx of adverse drug rx have very wide CIs.

Please explain the steps taken when performing the regression analysis.

**Discussion:**

1. Please state the corresponding figures obtained in at least one other country.

"Similar studies conducted in Europe,18,26 Asia,15 and Africa16,19 also reported a high prevalence of adverse reactions to COVID-19.

2. Any major reaction reported at all? How does the state deal with major, if any? Eg. yellow reporting form, pharmacovigilance processes.

3. Study limitation - if only one type then mention consistently throughout, objective, methods, results, etc. Consider adding to title too.

One major limitation of this study is the fact that all the respondents received only the AstraZeneca vaccine, thus it was not possible to compare the rate of adverse events across all the vaccines approved for the COVID-19 vaccination.

**Conclusion:**

Remove all the subheadings after conclusion. The contents could be organised as a separate para in the discussion (after strengths and limitation)

**Kindly provide evidence that proofreading was performed.**

**Kindly refer to PLOS guidelines for formatting of references.**
