# Supplementary material for: Prevalence and pattern of adverse events following COVID-19 vaccination among adult population in Sokoto metropolis, northwest, Nigeria
Source: PLoS One. 2025 Mar 12;20(3):e0277585. doi: 10.1371/journal.pone.0277585 (PMC11903040; doi:10.1371/journal.pone.0277585)
Supplement: Attachment — Submitted filename: Response to Reviewer - 3.docx [file pone.0277585.s010.docx]

**`REVIEWERS’ COMMENTS AND ACTIONS TAKEN BY AUTHORS - 3**

| SN | **COMMENTS/OBSERVATIONS OF REVIEWER** | **ACTION TAKEN** |
| --- | --- | --- |
|  | **Title** |  |
|  | 1. Suggested adding “an” to adult population | We thought adding “an” to it will suggest we are referring to a particular adult population in Sokoto state; however, our study did not focus on any specific/particular adult population, it considered all eligible adults who received COVID 19 vaccine from the selected health facilities |
| A | **Abstract** |  |
|  | 1. "As all vaccines are associated with some adverse reactions (ARs)" - to be removed from objectives. 2. Methods - COVID-19 vaccine - "s" to be added 3. Results: "M"ajority 4. Conclusion: Remove comma before ","with body weakness 5. | 1. Done 2. Done 3. Done 4. Done |
| B | **Introduction** | |
|  | 1. Corrections on lines 1 and 2 2. To consider removing paragraphs 2 and 3 3. To remove the phrases “problem statement”, “rationale”, hypothesis” 4. | 1. Done 2. Paragraph 3 removed 3. The phrases were not there in the initial version of the manuscript, but the authors were advised to include it in the previous review; the phrases have now been removed |
| C | **Materials and method** | |
|  | 1. Move around the contents so they fall under the specific subheadings. Use subheadings "Study design and sampling", "Study instruments", Inclusion and exclusion criteria", "Enrollment" 2. Please provide as supplementary material a completed checklist stating page numbers where each of the criterion was fulfilled https://www.strobe-statement.org/checklists/ 3. Rephrase so it reflects the past actions of the research team involved. "So if a researcher anticipates that up to 90% of the subjects will complete the questionnaires, he should divide the sample size by 90% (i.e. 0.9)." 4. The content under data collection subsection to be reorganised and made clearer. Currently unclear who made the calls, as there was a mention of research assistants in the preceding para. 5. Rephrase, "confidentiality of the information provided by the participants was also assured."   6. Study limitations to be moved to end of discussion section. | 1. The contents as presented herein are based on earlier corrections/suggestions by reviewers of the manuscript 2. Study designed explained;      3. In the previous review of the manuscript, the authors were asked to explain (with reference) their reason for adjusting the sample size to cater for non-response of the study participants   The past actions of the researchers are stated in the paragraph that followed the explanation   4. Done 5. Rephrased 6. Moved |
| D | **Result** | |
|  | 1. Please recalculate response rate using initial number eligible for inclusion, then excluded due to missing phone numbers, approached for interview but did not give consent etc 2. To refer tables 1 and 3 in the text 3. Figure 1 can be removed as it is described in text. 4. Rephrase "On bivariate analysis using Pearson chi square test 5. Logistic regression findings: both presence of comorbidities and hx of adverse drug rx have very wide CIs. Please explain the steps taken when performing the regression analysis. | 1. The initial number eligible for inclusion is 230 (based on the sample size calculated) and all those approached consented and responded to the questions. The only explanation was that, some study participants rescheduled in interview to a more convenient time for them. 2. Done 3. As the content of the chart is among the key objectives of the study, the researchers felt it would be better to present it in a chart because readers could miss it if presented in text only 4. Rephrased 5. The dependent variable was experience of AEFI following COVID 19 vaccination (coded as No-0 and Yes – 1). Three factors were found to have significant association with experience of AEFI, thus they were selected into the covariate box (after selecting regression in SPSS) as the independent variables (IV); experience of AEFI was selected into the Dependent variable box. Since all the 3 IV are categorical (with Yes/No options), they were further selected into the “Categorical Covariates” box with the last option (“No”) as the reference category. 95% CI was selected and the method used was “Enter” |
| E | **Discussion** | |
|  | 1. Please state the corresponding figures obtained in at least one other country.   "Similar studies conducted in Europe,18,26 Asia,15 and Africa16,19 also reported a high prevalence of adverse reactions to COVID-19”   2. Any major reaction reported at all? How does the state deal with major, if any? Eg. yellow reporting form, pharmacovigilance processes 3. Study limitation - if only one type then mention consistently throughout, objective, methods, results, etc. Consider adding to title too.   One major limitation of this study is the fact that all the respondents received only the AstraZeneca vaccine, thus it was not possible to compare the rate of adverse events across all the vaccines approved for the COVID-19 vaccination | 1. Done (a study from Mexico added) 2. Explanation given 3. Done |
| F | **Conclusion/recommendation** | |
|  | Remove all the subheadings after conclusion. The contents could be organised as a separate para in the discussion (after strengths and limitation) | Done |
| G | **Reference list** | |
|  | Nil |  |
|  | **Supporting document** | |
|  | To re-attach the file containing main data for the work | Done |
