# Supplementary material for: Prevalence and pattern of adverse events following COVID-19 vaccination among adult population in Sokoto metropolis, northwest, Nigeria
Source: PLoS One. 2025 Mar 12;20(3):e0277585. doi: 10.1371/journal.pone.0277585 (PMC11903040; doi:10.1371/journal.pone.0277585)
Supplement: Attachment — Submitted filename: Response to Reviewer - 5.docx [file pone.0277585.s011.docx]

**`REVIEWERS’ COMMENTS AND ACTIONS TAKEN BY AUTHORS - 5**

| SN | **COMMENTS/OBSERVATIONS OF REVIEWER** | **ACTION TAKEN** |
| --- | --- | --- |
|  | **Title** |  |
|  |  |  |
| A | **Abstract** |  |
|  |  |  |
| B | **Introduction** | |
|  | 1. Authors should explicitly state the hypothesis and primary objective of the study | 1. Done |
| C | **Materials and method** | |
|  | 1. The authors should detail the inclusion and exclusion criteria for the sample more comprehensively. For example, specify the exact criteria for inclusion (e.g., age range, health status) and exclusion (e.g., specific medical conditions, previous severe reactions to vaccines). 2. The authors should provide a precise definition and examples of what constitutes an AEFI in the context of this study. 3. The timeline for data collection is not specified 4. The authors claim that their data is representative of the entire population, but this assertion needs justification. They should explain why the chosen sample and methods are representative of the broader population of Sokoto Metropolis, considering demographic and geographic diversity. Authors should also discuss the possibility of sampling bias due to the selection of participants from specific health facilities and the use of phone interviews, which may exclude those without access to phones or those who are less willing to participate. 5. Since the data rely on self-reported information collected via phone calls, there is a risk of recall bias. Participants might not accurately remember or report their experiences, especially if significant time has passed since their vaccination. 6. Authors should reiterate the importance of addressing the study's limitations and suggest directions for future research | 1. Done 2. Done 3. The timeline has already been specified in the manuscript (page 9); see the last sentence under the subsection on questionnaire administration 4. At the time of collecting the data for the study, very few health facilities were providing COVID 19 vaccination service. Therefore, all recipients of the vaccine within the metropolis must access the service from one of these health facilities irrespective of their gender, race/ethnicity, religion or social status; thus, the sociodemographic diversity within the metropolis is taken into account. The only tertiary hospital within the metropolis was the one selected for this study; likewise, the primary and secondary health facilities selected in this study were selected from the few remaining health facilities providing the vaccination service via a probability sampling technique. All categories of health facilities (primary, secondary and tertiary) providing COVID 19 vaccination services at the time were included in the sampling frame. Based on the recipients’ record in the COVID 19 registers at the selected HFs, nearly all the recipients had their phone numbers of their next of kin’s number recorded. 5. This was mentioned as part of the limitations of the study, nevertheless, we believe their recall was good enough since all the adverse reactions were mentioned to the respondents during the phone call interviews. 6. Done |
| D | **Results** | |
|  |  |  |
| E | **Discussion** | |
|  |  |  |
| F | **Conclusion/recommendation** | |
|  |  |  |
| G | **Reference list** | |
|  | Nil |  |
|  | **Supporting document** | |
|  |  |  |
